# Liver Transplantation in a Myopathic Patient with Glycogen Storage Disease Type IIIa and Decompensated Cirrhosis

**Published:** 2017-11-01

**Authors:** M. Zobeiri

**Affiliations:** Department of Internal Medicine, Imam Reza Hospital, Kermanshah University of Medical Sciences, Kermanshah, Iran

**Keywords:** Cirrhosis, Glycogen storage disease types III, Liver transplantation, Liver diseases, Liver cirrhosis

## Abstract

Glycogen storage disease (GSD) type IIIa (Forbes-Cori disease) can be associated with severe liver disease. A patient with GSD type IIIa may therefore be a potential candidate for liver transplantation. Progressive myopathy makes uncertain the outcome of the patient and the transplant. Herein, we report on the good results of liver transplantation up to 28 months after the transplantation in a 40-year-old man with liver cirrhosis and significant muscle weakness due to GSD type IIIa.

## INTRODUCTION

Glycogen storage disease type III (GSD III) is a rare autosomal recessive hepatic disease characterized by glycogen accumulation due to defect in the gene AGL with deficiency of glycogen debranching enzyme activity that converts glycogen to glucose [1]. Glycogen accumulation occurs primarily in the liver and in skeletal and cardiac muscles with systemic consequences [2]. GSD III accounts for approximately 24% of GSDs with an estimated incidence of 1 case per 83,000 live births in Europe and 1 in 100,000 in North America [2, 3]. Four subtypes of GSD III, based on differences in tissue expression of the deficient enzyme, are recognized [4]. Relatively, 85% of GSD III is GSD IIIa subtype (Forbes-Cori disease) that involves both liver and muscle; 15% are of type IIIb, which have only liver involvement [5]. Two other subtypes, GSD IIIc and GSD IIId, are extremely rare [5]. Faroe Islands have the highest prevalence of GSD IIIa [6]. This relatively mild form of GSD presents in childhood and has a good prognosis [7]. Typical features include hepatomegaly that gets smaller with time, hypoglycemia, poor growth with eventual catch-up, skeletal myopathy that gradually worsens with time, and cardiomyopathy [7]. Hepatomegaly, hypoglycemia, hyperlipidemia, and growth retardation usually improve with age and disappear after puberty [4]. Muscle weakness in GSD IIIa is minimal during childhood; however, it can become predominant in adulthood and show signs of neuromuscular involvement with slowly progressive weakness and distal muscle wasting [6]. Long-term complications in adults mainly related to the muscles, heart and liver involvement. Hypertrophic cardiomyopathy usually develops during childhood in the majority of patients with GSD IIIa, but the seriousness of signs and symptoms varies from asymptomatic in the majority to severe heart disease and rarely heart failure [7]. Liver cirrhosis is a rare complication of GSD III [8]. There is no specific treatment for GSDs, but diet therapy with high-protein, frequent meals with corn starch supplements or nocturnal gastric drip feeding, constitutes effective therapy and improves hypoglycemia, liver size, and overall growth and development [2]. The detection of the molecular defect has facilitated the diagnosis and has offered the opportunity for prenatal diagnosis in these patients [9]. Currently, there is no effective treatment for the progressive myopathy or cardiomyopathy [4]. Diabetes mellitus (DM) in GSD III is difficult to treat because patients are prone to hypoglycemia [4]. Herein, we report on a case of GSD IIIa presenting with great skeletal myopathy and end-stage cirrhosis without hypoglycemia and cardiomyopathy that underwent liver transplantation (LT).

## CASE REPORT

A 40-year-old man presented with gastrointestinal bleeding due to esophageal varices and liver cirrhosis. Review of his records revealed that he had been diagnosed with GSD at the age of two years following a liver biopsy, when he had a distended abdomen due to hepatomegaly. He had problem with weakness from early childhood. In his childhood, he had frequent nose bleeding and easy fatigability. His growth was very slow. He was on no specific therapy and never suffered from symptomatic hypoglycemia. He went into a late puberty at about 17–18 years, but grew very rapidly and became much taller than his parents. During that period, his muscle weakness worsened and he had been in a wheelchair since he became 24 years old. His weakness in his arms also dated back to his puberty. At the time of presentation, he was unable to brush his hair and had to lean forward to eat. He had not had any heart problem. He had not had any sleep problems or chest infections. He suffered from low back pain after sleeping. His diet was high in protein with lots of meat, nuts, eggs, and yoghurt. He had never tried uncooked corn starch. GSD IIIa, for the first time, was confirmed at the age of 28 in the National Hospital for Neurology and Neurosurgery, Queen Square in London, on April 7, 2004. His parents were unrelated, alive and doing well. There was no family history of other muscle disorders. He had no other medical problems. He had normal schooling and studied engineering at university but had to stop in his 3^rd^ year because of the weakness. He was on no regular medication. He was unable to sitting in wheelchair and needed assistance to be transferred. On physical exam, he appeared dysmorphic, thin, disable but intelligent. His vital signs, chest and heart were normal without any evidence of cardiomyopathy. Abdominal examination revealed a firm and enlarged liver, splenomegaly, and ascites. Marked proximal and distal weakness, not involving face, was detected. The patient was unable to lift his arms or legs against the gravity. No deep tendon reflexes were presented. Muscular enzymes were elevated; muscle biopsy performed at age of 30 years. Because of massive upper gastrointestinal bleeding due to grade 4 esophageal varices, ascites, low platelet count, and fatigue, despite sever muscle weakness with weight, height and BMI of 56 kg, 180 cm, and 17.2 kg/m^2^, LT was performed. On 28^th^ month after transplantation, the patient had normal liver function and metabolic control without any restrictions and some weight gain up to 60 kg. However, he still had muscle weakness with elevated muscle enzymes. He was treated with prednisone 5 mg, mycophenolate 1500 mg, and tacrolimus 4 mg for prophylaxis of graft rejection. 

## DISCUSSION

This is the first report of patient with GSD IIIa who underwent LT in the middle-age, because of cirrhosis and variceal hemorrhage as an index complication despite loss of muscle mass and strength. The first report on LT in GSD III performed in 1989 [10]. GSD IIIa involves muscle and muscle weakness is mostly minimal at childhood, however, it progresses slowly and becomes prominent during the third and fourth decades of life [11]. Proximal muscles are primarily involved, but distal muscle wasting including the calves, hands, and peroneal muscles are also involved [11, 12]. Liver fibrosis is also a common feature of GSD III, and micronodular cirrhosis in some cases, has been found but appears not to progress in most instances. Hepatic adenoma or hepatocellular carcinoma has been reported in some cases [13]. LT is indicated when end-stage cirrhosis or hepatic malignancy is detected, but it has also been used with success in patients who are recalcitrant to medical therapy [14]. Ultimately, GSD IIIa is a multi-system disorder, and long-term success of LT, particularly with regard to myopathy or cardiomyopathy, is not known [15]. LT may adversely affect the symptoms of myopathy and cardiomyopathy [16]. 

Matern and colleagues demonstrated post-transplantation correction of metabolic abnormalities [5]. LT is a life-saving measure for patients with chronic end-stage liver disease and supposed to correct the primary hepatic enzyme defects and the deleterious complications of GSD with acceptable outcome and good long-term results [14, 17]. The extra-hepatic manifestations of GSD often complicate post-transplantation period [18]. LT is contraindicated for patients who are unlikely to survive the procedure or receive long-term benefits. Patients are considered individually and their candidacy is assessed by a formal multidisciplinary evaluation process [19]. LT does not cure the heart and muscle problems; transplantation has been associated with worsening myopathy and cardiomyopathy [20]. No systematic study has analyzed the risk factors for neuromuscular complications in LT. High doses of corticosteroids and the use of non-depolarizing neuromuscular blocking agents are reported to favor quadriplegia after LT [21]. Use of prednisone with dose of <10 mg/day is rarely associated with glucocorticoid-induced myopathy [22]. Therefore, discontinuation of prednisone probably has little effect on muscle weakness.
